# Refeeding Syndrome in Pediatric Age, An Unknown Disease: A Narrative Review

**DOI:** 10.1097/MPG.0000000000003945

**Published:** 2023-09-14

**Authors:** Antonio Corsello, Chiara Maria Trovato, Valeria Dipasquale, Giulia Bolasco, Flavio Labriola, Frédéric Gottrand, Elvira Verduci, Antonella Diamanti, Claudio Romano

**Affiliations:** From the * Department of Pediatrics, Vittore Buzzi Children’s Hospital, University of Milan, Milan, Italy; † Department of Health Science, University of Milan, Milan, Italy; ‡ Hepatology Gastroenterology and Nutrition Unit, Bambino Gesù Children Hospital, Rome, Italy; § Pediatric Gastroenterology and Cystic Fibrosis Unit, Department of Human Pathology in Adulthood and Childhood “G. Barresi,” University of Messina, Messina, Italy; ∥ Pediatric Gastroenterology Unit, Maggiore Hospital, Bologna, Italy; the ¶ Department of Pediatric Gastroenterology, Hepatology, and Nutrition, CHU Lille, University of Lille, Lille, France.

**Keywords:** anorexia nervosa, hypophosphatemia, micronutrients, neonatology, pediatric malnutrition, preterm newborns, refeeding syndrome, thiamine deficiency, weight loss

## Abstract

Refeeding syndrome (RS) is characterized by electrolyte imbalances that can occur in malnourished and abruptly refed patients. Typical features of RS are hypophosphatemia, hypokalemia, hypomagnesemia, and thiamine deficiency. It is a potentially life-threatening condition that can affect both adults and children, although there is scarce evidence in the pediatric literature. The sudden increase in food intake causes a shift in the body’s metabolism and electrolyte balance, leading to symptoms such as weakness, seizures, and even heart failure. A proper management with progressive increase in nutrients is essential to prevent the onset of this condition and ensure the best possible outcomes. Moreover, an estimated incidence of up to 7.4% has been observed in pediatric intensive care unit patients receiving nutritional support, alone or as an adjunct. To prevent RS, it is important to carefully monitor feeding resumption, particularly in severely malnourished individuals. A proper strategy should start with small amounts of low-calorie fluids and gradually increasing the calorie content and amount of food over several days. Close monitoring of electrolyte levels is critical and prophylactic use of dietary supplements such as thiamine may be required to correct any imbalances that may occur. In this narrative review, we aim to provide a comprehensive understanding of RS in pediatric clinical practice and provide a possible management algorithm.

## Graphical abstract

**Figure:**
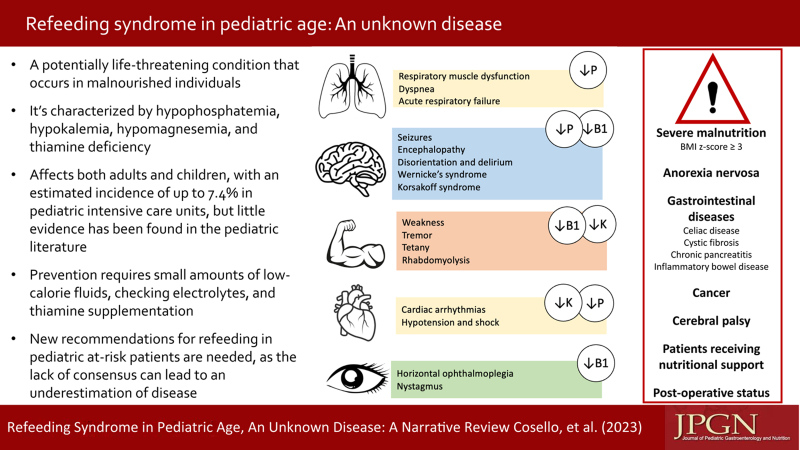

**What Is Known**

- Refeeding syndrome (RS) is a potentially life-threatening condition that can occur in malnourished individuals who resume feeding suddenly.
- RS is characterized by electrolyte imbalances, particularly hypophosphatemia, hypokalemia, hypomagnesemia, and thiamine deficiency.
- A gradual increase in calorie intake and a daily close monitoring of electrolyte levels are essential to prevent RS and ensure the best possible outcomes.

**What Is New**

- RS can affect both adults and children, but scarce evidence has been reported in pediatric literature.
- This narrative review provides a comprehensive understanding of RS, emphasizing the need for new pediatric recommendations to prevent its underestimation in at-risk patients and suggesting a possible algorithm of management.

Refeeding syndrome (RS) is a potentially life-threatening condition that can occur in malnourished adults and children who resume feeding suddenly and rapidly (1). This disease is characterized by a rapid shift in electrolyte and fluid balance that can cause a range of symptoms and complications, including cardiac arrhythmia, shortness of breath, seizures, and even death (2). RS is a complex interplay of metabolic and electrolyte imbalances that can lead to serious complications in various organs of the body (3). The human body relies on lipid and protein storages during malnutrition or starvation, leading to depletion of carbohydrate stores, mainly glycogen, in the liver and muscles (4). Re-eating, especially carbohydrates, raises insulin levels, which promote glucose uptake and utilization by cells, eventually resulting in rapid changes in electrolyte and fluid balance (5). Potassium, magnesium, and phosphate enter the cell, thereby causing a reduction of these electrolytes’ levels in the blood. The resulting electrolyte and fluid imbalances can cause various symptoms and complications (6). Hormonal and metabolic changes can also lead to changes in the acid-base balance in the body, causing metabolic alkalosis (7).

RS in adolescents and young adults is generally associated with marked malnutrition, mainly anorexia (8–10). However, several clinical conditions at different life stages pose a risk for RS (11–13). Recent reports describe an increased risk of developing RS in children and occurring in those with gastrointestinal diseases such as celiac disease and Crohn disease (14–17). According to the American Society for Parenteral and Enteral Nutrition (ASPEN), criteria for diagnosing RS include a 10%–20% drop in serum phosphate, potassium, and/or magnesium levels in mild RS and 20%–30% in moderate RS or greater than 30% and/or the presence of organ dysfunction due to a decrease in either of these electrolytes and/or thiamine deficiency in severe RS (18). In addition, these criteria should be met within 5 days of resuming or significantly increasing energy supply. In this review, we focus on the pathophysiology, clinical manifestations, risk factors, and current evidence for RS in neonatology and pediatrics. Furthermore, by examining the diagnostic criteria, we aim to provide a comprehensive understanding of the identification and classification of RS in clinical practice and provide a possible management algorithm.

## METHODS

The literature search for this narrative review was conducted in 3 databases (PubMed-Medline, Embase, and Web of Science) identifying the most relevant studies, reviews, and guidelines published up to March 2023 on RS and specifically its evidence and prevalence in pediatrics population ages have been published. The search was performed using combinations of the following keywords: refeeding syndrome, pediatric malnutrition, hypophosphatemia, anorexia nervosa, severe inflammatory bowel disease, parenteral nutrition, enteral nutrition, micronutrients, thiamine deficiency, marasmus, chronic fasting, and weight loss. Two reviewers independently reviewed the titles and abstracts of the identified studies to determine their relevance to the research topic.

## PATHOPHYSIOLOGY AND CLINICAL MANIFESTATIONS

**FIGURE 1.**
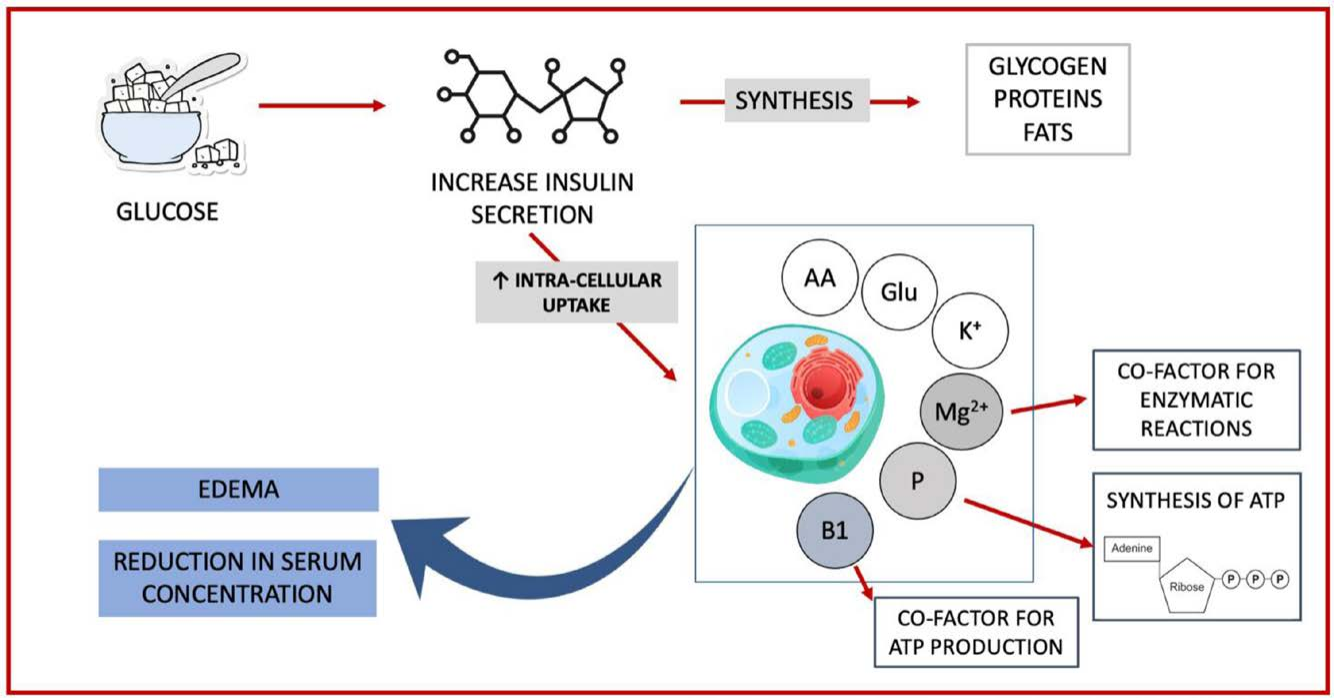
Pathophysiology of water/electrolyte imbalance in refeeding syndrome.

The pathophysiology of RS is not completely understood, although it is acknowledged that multifactorial mechanisms are present in this disease (19). During periods of prolonged starvation, the body adjusts to a catabolic state, relying on stored glycogen, fats, and eventually proteins for energy provision. Refeeding initiates a process of anabolism that requires increased energy and nutrient utilization for tissue repair and synthesis (20). However, in individuals with chronic malnutrition, the human body has adapted to a reduced metabolic rate and decreased nutrient utilization (21). In the event of sudden nutrient intake, our metabolism may not be able to meet metabolic needs, resulting in serious metabolic and electrolyte imbalances. When food is eaten again, especially in the form of carbohydrates, insulin levels rise, leading to an increase in glucose uptake and utilization (22). This chain results in a rapid shift in electrolyte and fluid balance with increased cellular uptake of potassium, magnesium, and phosphate, resulting in serum depletion of these electrolytes (23). Figure 1 attempts to summarize the pathophysiology of RS in the human body.

### Macronutrients

Under normal circumstances, the body uses glucose as its primary source of energy, which requires an adequate intake of carbohydrates. Two to three hours after carbohydrate intake, glucose is available and stored as glycogen (24). The amount of glycogen is limited and provides the body with a short-term energy source when no food is being consumed. Thus, the body retains proteins that would not normally be used for energy purposes (25). Excess calorie intake is generally stored in the form of fats, which are the human body’s main energy reserve (26). After a short fasting phase (approx. 24 hours), glycogenolysis in the liver and muscles compensates for the glucose deficiency. After glycogen is used up, gluconeogenesis begins. Amino acids from muscle proteins and fatty acids from adipose tissue provide the body with glucose as an energy source through metabolic reconstruction (27). Pyruvate and lactate can also provide glucose through gluconeogenesis. This fasting period is characterized by increased protein breakdown. Continued fasting slows down the body and reduces its basal metabolism by about 20%–25% (28). In these diseases, most organs and tissues rely on fatty acids as a source of energy. The brain is the organ that primarily uses glucose and can only partially switch to ketones for energy (29). By switching to fat as an energy source in the body, protein and muscle mass are preserved. During this period, there is a decrease in the rate of proteolysis, an increase in the migration of fatty acids and the formation of ketones. In addition, there is a reduction in intracellular concentrations of micronutrients, and although serum electrolyte may not change, this could be related to a contraction of intracellular space, decreased renal excretion, and a lack of body stores (1,2). In the attempt to preserve survival, the body implements the breaking down of its structural components in order to obtain energy substrates. Even if malnourished patients may be particularly affected by this metabolic change, RS can also occur in other people after prolonged fasting (30).

### Micronutrients

During the refeeding process, the increase in blood glucose leads to an increase in insulin secretion and a decrease in glucagon secretion (1). This hormonal shift induces the synthesis of glycogen, proteins, and fats (31). Minerals such as phosphorus and magnesium as well as cofactors such as thiamine are required for these processes. Insulin stimulates the uptake of potassium and glucose into the cell, and magnesium and phosphate also enter the cell (32). This leads to a reduction in serum concentrations of phosphates, potassium, and magnesium, which are generally common in already malnourished patients. The clinical symptoms of RS arise from the deficiency of these minerals and rapid changes in their serum concentrations leading to metabolic and electrolyte disturbances. The rapid drop in serum micronutrient levels during refeeding is believed to be a key factor in the development of RS (33). Deficiencies in these minerals and vitamins can lead to metabolic and electrolyte imbalances and contribute to worsening symptoms.

#### Phosphorus

Phosphorus, a primary intracellular mineral, is required for energy metabolism, nucleotide synthesis, and cell membrane function (34). It is present in the blood as free inorganic phosphate and protein-bound phosphate. It is essential for many intracellular processes, such as activating enzymes and messengers, storing energy as adenosine triphosphate (ATP), regulating the oxygen affinity of hemoglobin in tissues, and aiding in the repair of DNA, RNA, and cell membranes. During starvation serum phosphate levels decrease due to increased renal excretion and decreased intestinal absorption. In RS, phosphorus is depleted throughout the body and insulin secretion leads to increased cellular absorption and utilization of phosphate (35,36). In addition, intracellular phosphorus imbalance occurs when the body breaks down muscle tissue to provide energy through ATP consumption (37). Upon resumption of nutrition, the sudden increase in insulin secretion stimulates the cellular uptake of glucose, amino acids, and minerals such as phosphorus. However, if serum phosphate levels are already low, this rapid shift can worsen hypophosphatemia, which may result in typical RS symptoms (38). Under these conditions, even a small drop in serum phosphate levels leads to significant dysfunction of cellular processes affecting virtually every physiological system.

#### Magnesium and Potassium

In addition to phosphorus, deficiencies in other minerals such as magnesium and potassium can also contribute to the development of RS (23,32). Magnesium is an intracellular cation and a cofactor in most enzymatic systems (eg, oxidative phosphorylation, ATP production) (39). Its deficiency can lead to changes in neuromuscular excitability and heart disease. In addition to RS, other causes of acute hypomagnesemia can also be diarrhea, pancreatitis, malnutrition, alcoholism, metabolic acidosis, or specific therapies (eg, amphotericin B, furosemide, aminoglycosides, cisplatin, cyclosporin) (40). Magnesium is required for enzymatic reactions involved in energy metabolism, protein synthesis, and nucleotide synthesis. Hypomagnesemia is typically associated with hypocalcemia and hypokalemia because it activates the Na^+^/K^+^-ATPase pump and alters parathyroid hormone release (41). Potassium is needed for normal cell function and is an important electrolyte involved in muscle and nerve function (42). The onset of anabolic processes leads to potassium uptake into the insulin-stimulated cell, resulting in severe hypophosphatemia, changes in the electrochemical potential of the membrane, arrhythmias, and heart failure (43). Other contributory causes of hypokalemia, in addition to post-nutrition, can be diarrhea, increased urine leakage, metabolic alkalosis, or medication (eg, diuretics, adrenergic agents, glucocorticoids, insulin) (44).

#### Thiamine

Thiamine deficiency is the most common vitamin deficiency that occurs as a complication of RS (45,46). Thiamine is an essential cofactor of enzymes involved in carbohydrate metabolism and ATP synthesis (47). Its deficiency is typically known in Wernicke encephalopathy (ataxia, ophthalmoplegia, confusion, hypothermia, coma) and Korsakoff syndrome (amnesia, confabulation). Thiamine deficiency is the most common vitamin deficiency that occurs as a complication of RS (45,46). Thiamine is an essential cofactor of enzymes involved in carbohydrate metabolism and ATP synthesis (47). Its deficiency is typically known in Wernicke encephalopathy (ataxia, ophthalmoplegia, confusion, hypothermia, coma) and Korsakoff syndrome (amnesia, confabulation) (48). Although all vitamin deficiencies can occur to varying degrees with inadequate intake, thiamine is of paramount importance when post-meal complications are considered. Thiamine is an essential coenzyme in carbohydrate metabolism and acts as the main cofactor of pyruvate dehydrogenases in glycolysis (49). Thiamine deficiency can lead to encephalopathy-like symptoms such as ataxia, eye disorders, amnesia, and coma in these patients (46).

## RS IN NEONATOLOGY

The majority of RS cases affect children and adults. Nevertheless, current studies show a neonatal disease with comparable metabolic disorders (50–53). The first articles identifying neonatal RS appeared in 2013, and since then there has been an increasing number of articles addressing severe hypophosphatemia, or RS (54). As early and higher doses of parenteral amino acids have become standard practice, hypophosphatemia and other electrolyte and mineral abnormalities can occur if electrolyte and mineral deficiencies are present in the first few days of life (51–53). This may be because the cells are in an early anabolic state, which increases the uptake of phosphorus and potassium and releases calcium from bone tissue. In 1993, ASPEN issued its first recommendation, recommending 3 g/kg/day of amino acids in parenteral nutrition for low-birth-weight babies weighing less than 2500 g 1–2 days after delivery (55). As the importance of early parenteral nutrition became clear, particularly in the very low birth weight (VLBW) population, higher initial amino acid targets of 3.5–3.85 g/kg/day on the first day of life were recommended (56). To date, the administration of initial amino acid levels of 3–4 g/kg/day is the standard of care, as multiple studies have found greater nutritional benefits for ectopic growth and development (57). Since the concentration of certain amino acids in VLBW infants tends to decrease after the umbilical cord is cut, early protein supply in preterm infants is crucial to prevent negative nitrogen balance (58). Early amino acid supplementation reduces protein catabolism, increases insulin production, and improves glucose tolerance.

These benefits result in increased growth, which is critical for patients with short gestational age (SGA) and intrauterine growth restriction (IUGR) due to the need for catch-up growth (58). Therefore, health care professionals must adhere to these principles while balancing increased amino acid supplies with adequate phosphate supplies. Some algorithms have been proposed to ensure adequate phosphorus and calcium are present alongside early amino acid administration (51). Parenteral adjustment of the calcium and phosphorus ratio, particularly 1 mmol:mmol, can reduce hypercalcemia and hypophosphatemia after higher early doses of amino acid administration (23.5 g/kg/day) (59). After the first day of life, personalized amounts may be required if serum electrolytes are closely monitored (60). The overall incidence of RS in infants is unclear. Due to the dietary and therapeutic measures, however, fluctuations are to be expected. According to the ASPEN consensus paper, SGA, IUGR due to maternal comorbidities, elevated high umbilical artery resistance index (UA-RI), severe prematurity, VLBW or extremely low birth weight (ELBW), and a *z* score >2 are examples of traits that put newborns at risk of RS (18). Indeed, the incidence of RS-induced hypophosphatemia appears to be higher in IUGR neonates than in correctly developing VLBW infants (approximately 40% vs 9%) (61). It has also been reported that maternal preeclampsia was associated with a higher incidence of hypophosphatemia at birth, regardless of IUGR status (54). IUGR mimics a state of malnutrition during pregnancy (62). Placental insufficiency is the most common cause of IUGR and leads to a chronic state of fetal malnutrition, including deficits in muscle mass-to-body weight ratio, glycogen storage in liver and skeletal muscle, and adipose tissue (63). In addition, active potassium and phosphorus transport across the placenta may be reduced. Preterm IUGR newborns have lower muscle weight, lower glycogen storage, and adipose tissue, as well as lower bone mineralization and lower calcium and phosphorus stores (64). Igarashi et al (13) found hypophosphatemia in 15 of 49 (36%) VLBW infants in the first postnatal week.

Hypophosphatemia was significantly associated with UA-RI, meaning it could be a useful predictor of future development of RS-like hypophosphatemia in very low birth weight infants (13). A prospective cohort study conducted in 6 neonatal intensive care units found a 20% incidence of RS in ELBW infants (birth weight <1000 g), with increased morbidity and mortality (65). Studies and case reports of RS in newborns varies widely by population, dietary standards, description of the electrolyte abnormality, and treatment and management approaches (61). Although hypophosphatemia is the most common sign of RS in newborns, it is not well characterized. In addition, it is difficult to distinguish clinical signs of neonatal RS from prematurity problems. A recent systematic analysis includes 16 studies from 1946 to 2020 with 3688 infants (61). The incidence of hypophosphatemia (20%–90%), hypokalemia (8.8%–66.7%), and hypomagnesemia (1%–8.3%) varied between studies. There were significant differences between studies regarding the definition of hypophosphatemia, patient population (eg, gestational age, early gestational age, IUGR), and initial diet (eg, initial amino acid intake, calcium and phosphate ratio). It is difficult to determine the overall incidence of RS in neonates (61). The impact of RS-associated electrolyte abnormalities on clinical outcomes in preterm infants is uncertain. Clinical outcomes associated with hypophosphatemia include longer duration of mechanical ventilation, development of bronchopulmonary dysplasia, and increased mortality (61). In one study, hypophosphatemia was associated with a higher risk of bronchopulmonary dysplasia, the need for mechanical ventilation and patent ductus arteriosus (54). On the other hand, hypophosphatemia does not appear to be associated with an increased risk of late sepsis.

In general, there is a lack of information on specific management and surveillance measures for neonatal RS, such as normal serum electrolyte concentrations and dosing regimens for electrolyte replenishment. In 2020, ASPEN issued guidelines for the prevention and treatment of RS in children and adults. However, data are not sufficiently extensive to provide specific recommendations for newborns (18). In parenteral nutritional support, earlier and adequate supplies of phosphorus and potassium and altered calcium and phosphorus ratios can reduce electrolyte imbalance and associated problems (52,54). Some authors have suggested enteral treatment as the safest method of electrolyte replacement (66). Enteral nutrition can be provided through the routine fortification of preterm milk or the use of preterm formula, as well as through the use of an enteral nutritional supplement in addition to normal nutrition. During the first week of life, in patients at risk, serum phosphate and electrolyte levels must be closely monitored, preferably via blood gas analysis, which requires less blood and generally provides immediate and more reliable results (67). In addition, early administration of phosphate in a molar ratio of 1:1 with calcium in the first week of life is recommended for patients with the highest RS risk (61).

## CURRENT EVIDENCE IN CHILDREN AND ADOLESCENTS

Several studies have been conducted in recent years to better understand the incidence, risk factors, and treatment strategies of RS in children. Recent studies conducted on pediatric patients (0–18 years) hospitalized in a single pediatric intensive care unit and receiving exclusive or supplemental nutritional support found that 15.8% of them were malnourished (68,69). Probable RS was diagnosed in 93 of these children, for an overall incidence of 7.4%, while 10.2% presented a body mass index *z* score decline greater than 1. The incidence rate in children at risk was 46.7%, and most patients (58.1%) had an ex-post diagnosis of severe RS. This study, involving a large population of 1261 children, underscores the importance of screening children for RS in high-risk contexts to adequately diagnose, prevent, treat, and monitor RS (68). Moreover, it underscores the importance of detecting and treating RS in pediatric patients, particularly those with chronic disease or malnutrition.

Main treatment strategies for RS in children involve a multidisciplinary approach with careful monitoring and supportive care to prevent or treat the complications of this condition. In addition, the treatment of eating disorders, and in particular anorexia nervosa, consists in the gradual restoration of weight and the prevention or treatment of clinical complications such as unstable vital signs, laboratory, and/or cardiac abnormalities. However, in spite of the availability of multiple guidelines, there is no clear consensus on precise evidence-based protocols (70–73). In fact, measures to restore body weight and prevent RS should be initiated carefully. Clinicians usually start with low-calorie supplementation, with subsequent slow rises. However, this approach of initiating with low calorie supplementation followed by slow increases has been questioned by some authors because it has been linked to low weight gain, as well as to malnutrition syndrome (74,75). In the systematic review by Garber et al (76) various approaches to in-hospital replenishment have been identified. Individuals with a higher caloric intake (>1400 kcal/day) were not associated with a higher risk of RS in patients with mild to moderate malnutrition. In contrast, in severely malnourished patients, there was no evidence in support of changes in current practice.

A recent retrospective chart analysis (77) aimed to compare lower-calorie and higher-calorie dietary approaches in adolescents and young adults with eating disorders at different stages of malnutrition (mean age 15.3 years). The low-calorie approach consisted of administering 30–40 kcal/kg of actual body weight (eg, 1000–1200 kcal) per day, with a maximum of 1000 kcal/day in severe malnutrition. The high-calorie strategy provided 1500–1700 kcal per day, except in severely malnourished patients whose daily caloric intake did not exceed 1200 kcal. The authors concluded that the length of hospital stay was significantly shorter in the high-calorie group and in patients with a higher mean body mass index, while prescribing higher calories was not associated with a higher rate of RS manifestations such as hypomagnesemia and hypokalemia. In contrast, a higher rate of hypophosphatemia was observed in the lower calorie intake group. Some authors have suggested the efficacy and safety of prophylactic oral phosphate supplementation in preventing RS among patients with restrictive eating disorders, but the evidence is still scant (78).

When planning nutritional treatment for eating disorders, the Society for Adolescent Health and Medicine recommends a 2-step process:

1. Assessing the degree of malnutrition using percent mean body mass index (BMI), *z* scores, and the extent and rate of weight loss.
2. Determining the healthy weight range of that individual patient based on previous height, weight, and BMI percentiles, pubertal stage, and growth trajectory (73).

## RISK FACTORS, EARLY IDENTIFICATION, AND PROPER MANAGEMENT

A crucial goal for the clinician, particularly when evaluating children, is to identify potentially at-risk patients. Both acute and chronic malnutrition are typical risk factors. In acute cases, weight loss of >10% in less than 3 months and/or 10–14 days of malnutrition (eg, in children receiving intravenous fluids without supplemental proteins/lipids or appropriately dosed electrolytes) result in increased risk (79). Common diseases that are responsible for chronic malnutrition (*z* score BMI <−2 SD) and increase risk of RS are anorexia nervosa, cancer, cystic fibrosis, or other chronic diseases that can cause malnutrition (1,5). The risk is even higher in people who have been malnourished for a long time, have lost significant weight, or are at increased risk of electrolyte imbalance (80). The lack of a clear definition of pediatric RS results in the absence of randomized controlled trials and published data on frequency. In addition, slight symptoms are often not recognized, and low levels of electrolytes can be secondary to other diseases. Therefore, the true prevalence of RS in children remains unknown. In adults, according to a study including 10,197 hospitalized patients, the overall incidence of severe hypophosphatemia was 0.43% (81). Results from other studies performed on adult patients receiving total parenteral nutrition found that up to 30%–43% of them suffered from hypophosphatemia, even when phosphate was included in the daily parenteral nutrition (82,83).

In addition, a frequency of RS in cancer has been observed in over 25% of patients (30). The population in intensive care units is then at high risk of RS given the high prevalence of malnutrition in this population (15%–25%) and the recommendation for early reintroduction of enteral nutrition (68). As already mentioned, according to the retrospective study by Blanc et al the absolute incidence was 7.4% in patients receiving supplemental nutrition and 46.7% in children at risk (BMI *z* score −2 SD). In addition, most patients (58.1%) were diagnosed with severe RS (68). Early identification of at-risk patients is a cornerstone of treatment. A gradual reintroduction of diet should be implemented to allow the body to adjust to the increased nutrient intake and reduce the risk of electrolyte imbalance (84). Daily close monitoring of electrolyte levels with serial blood gas analyses (and every 12 hours for the first 3 days) may be necessary, together with a proper dietary supplementation (18,23,33). Vitamin and mineral supplementation should also be considered. Fluid balance should be closely monitored, and diuretics may be needed to manage fluid overload (5). RS complications should be recognized and treated promptly. Nutritional support should be tailored to the individual needs of the patient, and psychosocial support should be provided to children and their families.

As said, the rapid insulin release in response to refeeding can lead to hypophosphatemia. Low blood phosphate levels may cause muscle weakness, impaired heart function, difficulty breathing, seizures, and coma. Hypophosphatemia in this situation can be managed by monitoring and correcting electrolyte imbalances, phosphate administration, gradual feeding, and vitamin supplementation (85).

RS symptoms are variable and unpredictable, reflecting the nature and severity of the underlying biochemical abnormality (22). The spectrum of symptoms can range from nausea and vomiting to respiratory failure, heart failure, hypotension, cardiac arrhythmias, coma, and death. Therefore, prevention and early detection of at-risk patients, monitoring during refeeding, and an appropriate diet regimen are key to successful treatment. Make-up should start at a maximum of 50% of the recommended energy requirement. In high-risk patients (chronic malnutrition, poor or no food intake for 10 days or more), nutritional restoration should be started even more slowly (10 kcal/kg/day or even less) and gradually increased to full requirement in about 7 days (18,32,82,86).

A possible algorithm of RS prevention should then include:

- Identification of high-risk patients:
  - Recent weight loss of 10% (or <80% of the ideal weight) receiving intravenous (IV) fluids only
  - Weight loss for 5 consecutive days, or poor fluid intake for more than 7 days
  - Chronic diseases causing malnutrition [eg, cancer, (inflammatory bowel disease IBD)], anorexia nervosa, marasmus, kwashiorkor
  - Prolonged fasting or low-energy diet
  - Hypoalbuminemia
- Calculation of resting energy expenditure (REE), the energy expended to maintain normal body function and homeostasis at rest, which could be estimated or measured. Indirect calorimetry is the most recommended measurement method, but it is often impractical in some clinical situations (87). Because of these limitations, predictive equations for estimating REE have been created and are mainly used in clinical practice (88–90).
- Complete blood and nutritional assessment including zinc, B12, folic acid, iron.
- Echocardiography and neurological examination.
- Before any type of refeeding, identify what supplements you need to prescribe.
- Correct any electrolyte imbalances before beginning the diet.

Daily thiamine supplementation is therefore crucial in both the treatment and prevention of RS, particularly in patients at increased risk of vitamin deficiencies. For example, in malnourished or alcoholic individuals, thiamine stores can become depleted, leading to potentially serious complications such as Wernicke-Korsakov syndrome. ASPEN recommends (18):

1. A thiamine dose of 2 mg/kg to a maximum of 100–200 mg/day prior to feeding or before beginning intravenous administration of dextrose-containing fluids in high-risk pediatric patients.
2. Continue thiamine supplementation for at least 5–7 days, or even longer, in patients at high-risk of RS.

**FIGURE 2.**
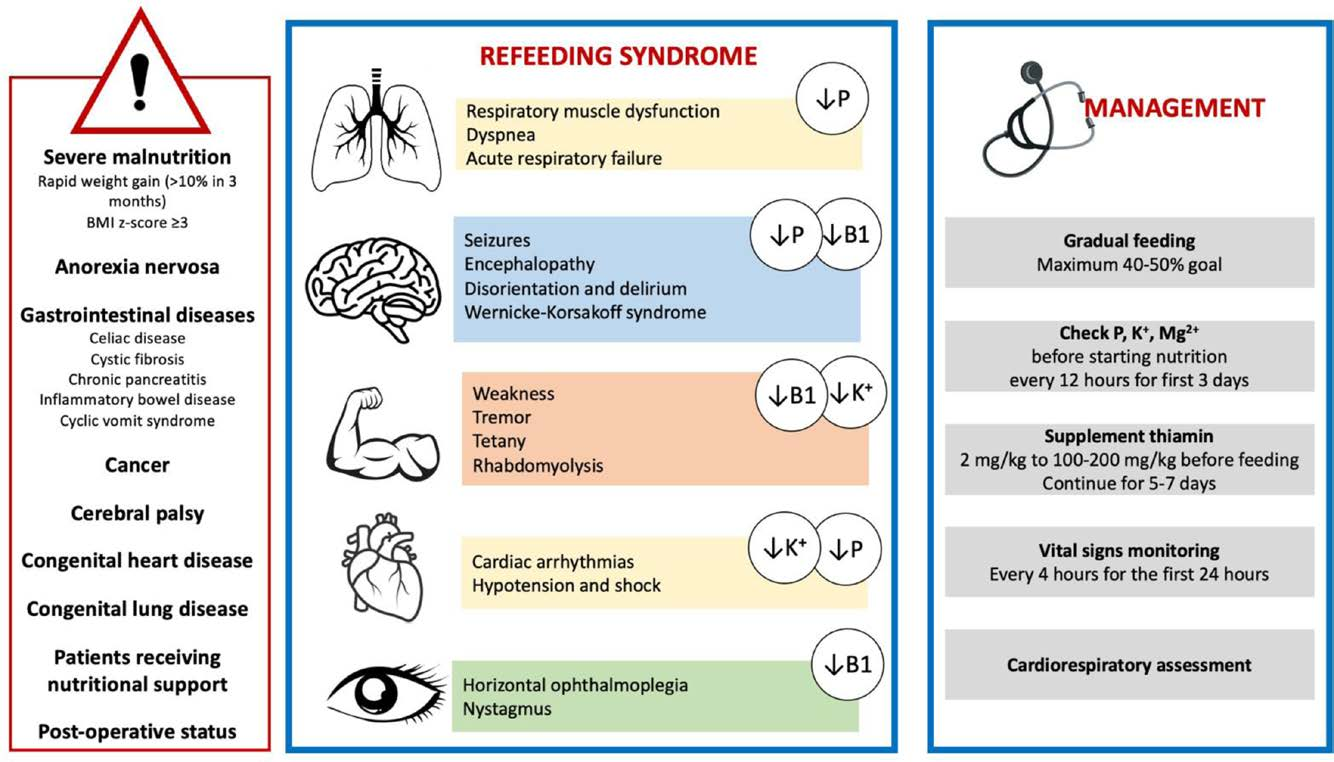
An overview of risk factors, symptoms, and proper management of refeeding syndrome.

Overall, management of pediatric RS requires close monitoring, careful attention to fluid and electrolyte balance, and a multidisciplinary approach to managing the various complications and challenges associated with this condition (Fig. 2). Further research is needed to better understand the risk factors, prevention, and treatment of this disease in pediatric age. Table 1 describes main strategies for use in at-risk patients with hypophosphatemia.

**TABLE 1.** Suggested treatment strategies of malnourished patients presenting hypophosphatemia

Strategy	Recommendation
Monitoring electrolyte imbalances and phosphate administration	Daily electrolyte levels monitoring is suggested. Phosphate administration may be necessary to treat severe hypophosphatemia. Depending on the severity of the disease and the patient’s tolerance, phosphate can be administered orally or intravenously. Because hypophosphatemia can be associated with other electrolyte imbalances, such as hypokalemia or hypocalcemia, these imbalances can be corrected to treat hypophosphatemia. Always consider potential drugs (eg, iron therapy) that could quickly and directly lower serum phosphate levels (91,92).
Gradual refeeding	To prevent hypophosphatemia from occurring, a gradual and progressive diet can be introduced. This strategy may be particularly important in patients at high risk for developing RS.
	Start nutrition at a maximum of 40%–50% goal, gradually increasing glucose infusion rate by 1–2 mg/kg/min daily until reaching a maximum of 14–18 mg/kg/min, including enteral and parenteral glucose (18).
Vitamin supplementation	Hypophosphatemia can be associated with micronutrient deficiencies, particularly vitamin B1 (thiamine). Therefore, thiamine supplementation is often recommended as part of the treatment and prevention of RS for at least 5–7 days (1,6,46). The recommended approach to treating refeeding syndrome in infants is to begin oral administration of 100 mg of thiamine, followed by starting infant formula at 50% of estimated needs. For infants, this typically corresponds to an intake of around 50–60 kcal/kg per day. Thereafter, calorie intake can be gradually increased by 25% per day (80).
	Supplementation with other vitamins may be necessary to treat hypophosphatemia and prevent associated complications. This recommendation is based on clinical experience and expert opinion, although there is limited high-quality evidence on the optimal dose and duration of thiamine supplementation for the prevention of RS.
Enteral nutrition	Give preference to enteral feeding whenever possible. Enteral nutrition in general has numerous advantages, particularly in malnourished patients, and is the preferred method over parenteral nutrition whenever possible (6,93). The recommended caloric and food intake should be by mouth, nasogastric tube, or other postpyloric feeding method (eg, PEG).
	The choice of route of administration should be individualized based on the needs of the patient and the availability of the formula. It is recommended that enteral nutrition be started as early as possible because parenteral nutrition carries a higher risk of infection and other complications and should be reserved for patients who cannot tolerate the necessary caloric intake via the enteral route (6,94). However, patients with obstructive symptoms, malabsorption disorders, or medical conditions that preclude enteral nutrition may require parenteral nutrition (95).

RS = refeeding syndrome.

## CONCLUSIONS

RS, particularly in vulnerable children, should be viewed as a potentially fatal condition caused by rapid refeeding after an indefinite period of malnutrition. Hypophosphatemia is characteristic, which is associated with fluid and electrolyte imbalances as well as metabolic and clinical complications. Awareness of pediatric RS and identification of at-risk patients is crucial, since this disorder is preventable, and metabolic complications are avoidable. High-risk patients include chronically malnourished patients and those who have had little or no energy for more than 10 days, who needs for daily monitoring of electrolytes levels. Refeeding should be started at a low level of energy replacement. Lastly, vitamin supplementation should be started with refeeding and continued for at least 5–7 days. Correction of fluid and electrolyte imbalances prior to feeding is not necessary and should be done at the same time as feeding.

To prevent RS, it is important to slowly reintroduce food and monitor the patient’s electrolyte levels. This can be accomplished by gradually increasing the number of calories in the diet and monitoring potassium, magnesium, and phosphate. If RS occurs, treatment may include correction of electrolyte imbalances with dietary supplements or intravenous fluids, and supportive measures such as oxygen therapy or mechanical ventilation as needed. Overall, RS is a serious but preventable condition that can be managed with careful monitoring and controlled treatment. At-risk patients should be closely monitored by health care professionals to ensure their safety and well-being. This review underscores the need to establish new pediatric recommendations for refeeding in at-risk patients, as the lack of consensus and pediatric guidelines can lead to an underestimation of RS.
